# 3-(Pyridin-4-yl­thio)­pentane-2,4-dione

**DOI:** 10.1107/S1600536811011330

**Published:** 2011-03-31

**Authors:** Qing-Fu Zhang, Jian-Dong Pang, De-Zhi Sun, Cai-Hua Liu

**Affiliations:** a College of Chemistry and Chemical Engineering, Liaocheng University, Liaocheng 252059, People’s Republic of China

## Abstract

In the title compound, C_10_H_11_NO_2_S, the acetyl­acetone group crystallizes in the keto form with all the non-hydrogen atoms in the acetyl­acetone group approximately co-planar with a maximum atomic deviation 0.055 (2) Å; the dihedral angle between the acetyl­acetone group and the pyridine ring is 85.90 (6)°. An intra­molecular O—H⋯O hydrogen bond involving the acetyl­acetone group forms a six-membered ring.

## Related literature

For applications of β-diketones and their derivatives in metallo-supra­molecular chemistry, see: Aromí *et al.* (2008 ▶); Chen *et al.* (2003 ▶; 2004 ▶); Domasevitch *et al.* (2006 ▶); Massue *et al.* (2005 ▶); Soldatov & Ripmeester (2001 ▶); Tabellion *et al.* (2001 ▶); Vigato *et al.* (2009 ▶); Vreshch *et al.* (2003 ▶, 2004 ▶); Won *et al.* (2007 ▶); Zhang *et al.* (2006 ▶).
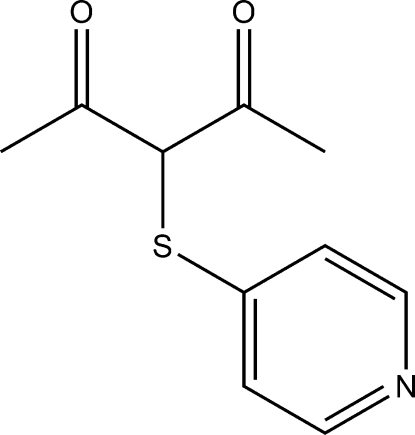


## Experimental

### 

#### Crystal data

C_10_H_11_NO_2_S
*M* *_r_* = 209.26
Monoclinic, 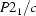

*a* = 8.3273 (7) Å
*b* = 9.5614 (8) Å
*c* = 13.0681 (11) Å
β = 92.698 (1)°
*V* = 1039.34 (15) Å^3^
*Z* = 4
Mo *K*α radiation
μ = 0.28 mm^−1^
*T* = 298 K
0.35 × 0.30 × 0.28 mm

#### Data collection

Siemens SMART CCD area-detector diffractometer
Absorption correction: multi-scan (*SADABS*; Sheldrick, 1996 ▶) *T*_min_ = 0.907, *T*_max_ = 0.925
5011 measured reflections
1822 independent reflections
1351 reflections with *I* > 2σ(*I*)
*R*_int_ = 0.030

#### Refinement

*R*[*F* ^2^ > 2σ(*F* ^2^)] = 0.038
*wR*(*F* ^2^) = 0.120
*S* = 1.06
1822 reflections
130 parameters
H-atom parameters constrained
Δρ_max_ = 0.17 e Å^−3^
Δρ_min_ = −0.19 e Å^−3^

### 

Data collection: *SMART* (Siemens, 1996 ▶); cell refinement: *SAINT* (Siemens, 1996 ▶); data reduction: *SAINT*; program(s) used to solve structure: *SHELXS97* (Sheldrick, 2008 ▶); program(s) used to refine structure: *SHELXL97* (Sheldrick, 2008 ▶); molecular graphics: *SHELXTL* (Sheldrick, 2008 ▶); software used to prepare material for publication: *SHELXTL*.

## Supplementary Material

Crystal structure: contains datablocks I, global. DOI: 10.1107/S1600536811011330/zj2007sup1.cif

Structure factors: contains datablocks I. DOI: 10.1107/S1600536811011330/zj2007Isup2.hkl

Additional supplementary materials:  crystallographic information; 3D view; checkCIF report

## Figures and Tables

**Table 1:** Hydrogen-bond geometry (Å, °)

*D*—H⋯*A*	*D*—H	H⋯*A*	*D*⋯*A*	*D*—H⋯*A*
O1—H1⋯O2	0.88	1.58	2.427 (3)	161

**Fig. 1.**
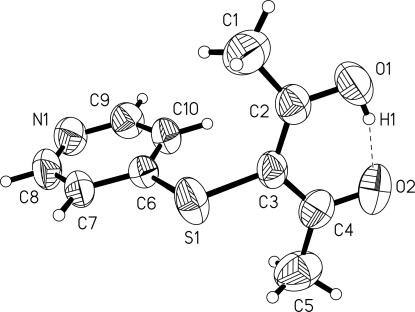
The molecular structure of the title compound, showing 50% probability displacement ellipsoids.

**Fig. 2.**
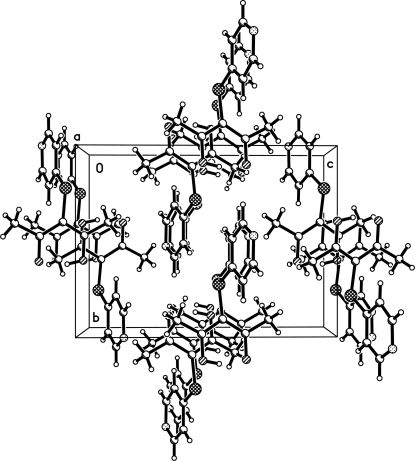
The packing diagram of the title compound.

## supplementary crystallographic information

### Comment

The β-diketones and their derivatives have attracted much attention in recent years, not only for their valuable intrinsic chemical and physical properties but also for their wide applications in metallo-supramolecular chemistry (Aromí & Gamez *et al.*,2008; Massue & Bellec *et al.*, 2005; Soldatov & Ripmeester, 2001; Tabellion & Seidel *et al.*, 2001; Vigato & Peruzzo *et al.*, 2009; Chen & Fronczek *et al.*,2003; Chen & Fronczek *et al.*,2004; Domasevitch & Vreshch, *et al.*, 2006; Vreshch & Chernega *et al.* 2003; Vreshch & Lysenko *et al.* 2004; Won & Clegg *et al.*,2007; Zhang & Chen *et al.*, 2006). Among them, the N-containing bifunctional derivatives of β-diketones have been successfully applied to construct various metal-organic supramolecular complexes (Chen & Fronczek *et al.*,2003; Chen & Fronczek *et al.*,2004; Domasevitch & Vreshch, *et al.*, 2006; Vreshch & Chernega *et al.* 2003; Vreshch & Lysenko *et al.* 2004; Won & Clegg *et al.*,2007; Zhang & Chen *et al.*, 2006). We report here on the structure of an interesting nonlinear N-containing bifunctional compound, 3-(pyridin-4-ylthio)pentane-2,4-dione, which is prepared according to the previously reported methord (Won & Clegg *et al.*,2007).

As shown in Fig. 1, the acetylacetone group is with keto-enol tautomerism, where all the non-hydrogen atoms in the acetylacetone group are approximately co-planar with a maximum atomic deviation 0.0547 (24) Å, and the dihedral angle between the acetylacetone group and the pyridine ring is about of 85.90 (6)°. The bond angle of C3—S1—C6 is about of 105.16 (10)°, leading to a V-shaped conformation of the whole molecule. In the crystal strucutre, the intramolecular O—H···O hydrogen bonding interactions have been found in the same acetylacetone group, forming a six-membered cyclic structure. However, no intermolecular H-bonding interactions have been found in the title comound (Fig. 2).

### Experimental

The title compound, 3-(pyridin-4-ylthio)pentane-2,4-dione, was prepared according to the previously reported method (Won & Clegg *et al.*,2007). The yellow crystals of title compound suitable for X-ray crystallographic analysis were obtained by recrystallization from acetonitrile.

### Refinement

All H atoms on C atoms were positioned geometrically and refined as riding atoms with d(C—H) =0.93–0.97 Å, and *U*_iso_(H) =1.2*U*_eq_(C). The H atom on O atom of enol group was located from difference Fourier map and refined with d(O—H) =0.881 Å, and *U*_iso_(H) =1.2*U*_eq_(O).

### Crystal data

**Table:**

C_10_H_11_NO_2_S	*F*(000) = 440
*M**_r_* = 209.26	*D*_x_ = 1.337 Mg m^−^^3^
Monoclinic, *P*2_1_/*c*	Mo *K*α radiation, λ = 0.71073 Å
Hall symbol: -P 2ybc	Cell parameters from 2174 reflections
*a* = 8.3273 (7) Å	θ = 2.5–27.3°
*b* = 9.5614 (8) Å	µ = 0.28 mm^−^^1^
*c* = 13.0681 (11) Å	*T* = 298 K
β = 92.698 (1)°	Block, yellow
*V* = 1039.34 (15) Å^3^	0.35 × 0.30 × 0.28 mm
*Z* = 4	

### Data collection

**Table:**

Siemens SMART CCD area-detector diffractometer	1822 independent reflections
Radiation source: fine-focus sealed tube	1351 reflections with *I* > 2σ(*I*)
graphite	*R*_int_ = 0.030
phi and ω scans	θ_max_ = 25.0°, θ_min_ = 2.6°
Absorption correction: multi-scan (*SADABS*; Sheldrick, 1996)	*h* = −9→9
*T*_min_ = 0.907, *T*_max_ = 0.925	*k* = −5→11
5011 measured reflections	*l* = −15→15

### Refinement

**Table:**

Refinement on *F*^2^	Secondary atom site location: difference Fourier map
Least-squares matrix: full	Hydrogen site location: inferred from neighbouring sites
*R*[*F*^2^ > 2σ(*F*^2^)] = 0.038	H-atom parameters constrained
*wR*(*F*^2^) = 0.120	*w* = 1/[σ^2^(*F*_o_^2^) + (0.0562*P*)^2^ + 0.3241*P*] where *P* = (*F*_o_^2^ + 2*F*_c_^2^)/3
*S* = 1.06	(Δ/σ)_max_ < 0.001
1822 reflections	Δρ_max_ = 0.17 e Å^−^^3^
130 parameters	Δρ_min_ = −0.18 e Å^−^^3^
0 restraints	Extinction correction: *SHELXL*, Fc^*^=kFc[1+0.001xFc^2^λ^3^/sin(2θ)]^-1/4^
Primary atom site location: structure-invariant direct methods	Extinction coefficient: 0.136 (9)

### Special details

**Table:**

Geometry. All e.s.d.'s (except the e.s.d. in the dihedral angle between two l.s. planes) are estimated using the full covariance matrix. The cell e.s.d.'s are taken into account individually in the estimation of e.s.d.'s in distances, angles and torsion angles; correlations between e.s.d.'s in cell parameters are only used when they are defined by crystal symmetry. An approximate (isotropic) treatment of cell e.s.d.'s is used for estimating e.s.d.'s involving l.s. planes.
Refinement. Refinement of *F*^2^ against ALL reflections. The weighted *R*-factor *wR* and goodness of fit *S* are based on *F*^2^, conventional *R*-factors *R* are based on *F*, with *F* set to zero for negative *F*^2^. The threshold expression of *F*^2^ > σ(*F*^2^) is used only for calculating *R*-factors(gt) *etc*. and is not relevant to the choice of reflections for refinement. *R*-factors based on *F*^2^ are statistically about twice as large as those based on *F*, and *R*- factors based on ALL data will be even larger.

### Fractional atomic coordinates and isotropic or equivalent isotropic displacement parameters (Å^2^)

**Table:**

	*x*	*y*	*z*	*U*_iso_*/*U*_eq_	
S1	0.87871 (8)	0.22005 (6)	0.95516 (6)	0.0688 (3)	
O1	0.7336 (2)	0.60208 (19)	1.01493 (15)	0.0761 (6)	
H1	0.7715	0.6213	0.9547	0.091*	
O2	0.8608 (2)	0.60632 (19)	0.85162 (14)	0.0769 (6)	
N1	0.4443 (3)	−0.0271 (2)	0.83293 (19)	0.0714 (6)	
C1	0.6930 (4)	0.3985 (4)	1.1103 (2)	0.0863 (9)	
H1A	0.7813	0.3688	1.1550	0.129*	
H1B	0.6305	0.3185	1.0886	0.129*	
H1C	0.6266	0.4623	1.1461	0.129*	
C2	0.7557 (3)	0.4691 (3)	1.01961 (19)	0.0566 (6)	
C3	0.8327 (3)	0.3980 (2)	0.94164 (17)	0.0495 (6)	
C4	0.8804 (3)	0.4736 (3)	0.85562 (18)	0.0580 (6)	
C5	0.9512 (4)	0.4065 (4)	0.7658 (2)	0.0890 (10)	
H5A	1.0051	0.4759	0.7269	0.133*	
H5B	0.8674	0.3639	0.7235	0.133*	
H5C	1.0270	0.3363	0.7889	0.133*	
C6	0.7057 (3)	0.1310 (2)	0.90682 (16)	0.0466 (5)	
C7	0.7102 (3)	−0.0134 (2)	0.90713 (19)	0.0584 (6)	
H7	0.8011	−0.0607	0.9327	0.070*	
C8	0.5786 (3)	−0.0856 (3)	0.8692 (2)	0.0730 (8)	
H8	0.5843	−0.1827	0.8690	0.088*	
C9	0.4412 (3)	0.1119 (3)	0.8347 (2)	0.0630 (7)	
H9	0.3474	0.1561	0.8107	0.076*	
C10	0.5674 (3)	0.1949 (2)	0.86962 (18)	0.0542 (6)	
H10	0.5591	0.2919	0.8680	0.065*	

### Atomic displacement parameters (Å^2^)

**Table:**

	*U*^11^	*U*^22^	*U*^33^	*U*^12^	*U*^13^	*U*^23^
S1	0.0565 (4)	0.0432 (4)	0.1043 (6)	0.0034 (3)	−0.0216 (3)	−0.0036 (3)
O1	0.0818 (13)	0.0532 (11)	0.0934 (14)	0.0072 (9)	0.0045 (10)	−0.0142 (9)
O2	0.0901 (14)	0.0563 (11)	0.0827 (13)	−0.0179 (10)	−0.0131 (10)	0.0166 (9)
N1	0.0674 (14)	0.0546 (13)	0.0923 (17)	−0.0155 (11)	0.0029 (12)	−0.0121 (11)
C1	0.090 (2)	0.095 (2)	0.0750 (19)	−0.0214 (18)	0.0168 (15)	−0.0053 (16)
C2	0.0498 (13)	0.0525 (14)	0.0668 (15)	−0.0059 (10)	−0.0058 (11)	−0.0064 (11)
C3	0.0459 (12)	0.0407 (12)	0.0610 (14)	−0.0075 (9)	−0.0053 (10)	−0.0053 (10)
C4	0.0484 (13)	0.0634 (16)	0.0614 (15)	−0.0137 (11)	−0.0080 (11)	−0.0028 (12)
C5	0.080 (2)	0.116 (3)	0.0717 (19)	−0.0176 (18)	0.0129 (15)	−0.0173 (17)
C6	0.0502 (13)	0.0403 (12)	0.0495 (12)	−0.0014 (9)	0.0039 (9)	−0.0023 (9)
C7	0.0605 (15)	0.0398 (12)	0.0751 (16)	0.0029 (10)	0.0060 (12)	0.0046 (11)
C8	0.076 (2)	0.0402 (13)	0.103 (2)	−0.0097 (13)	0.0085 (16)	−0.0015 (13)
C9	0.0570 (14)	0.0594 (16)	0.0720 (16)	−0.0022 (12)	−0.0043 (12)	−0.0048 (12)
C10	0.0577 (14)	0.0413 (12)	0.0630 (14)	−0.0010 (10)	−0.0044 (11)	−0.0041 (10)

### Geometric parameters (Å, °)

**Table:**

S1—C3	1.752 (2)	C4—C5	1.484 (4)
S1—C6	1.764 (2)	C5—H5A	0.9600
O1—C2	1.286 (3)	C5—H5B	0.9600
O1—H1	0.8814	C5—H5C	0.9600
O2—C4	1.280 (3)	C6—C10	1.373 (3)
N1—C8	1.318 (3)	C6—C7	1.381 (3)
N1—C9	1.330 (3)	C7—C8	1.368 (4)
C1—C2	1.480 (4)	C7—H7	0.9300
C1—H1A	0.9600	C8—H8	0.9300
C1—H1B	0.9600	C9—C10	1.378 (3)
C1—H1C	0.9600	C9—H9	0.9300
C2—C3	1.404 (3)	C10—H10	0.9300
C3—C4	1.409 (3)		
			
C3—S1—C6	105.16 (10)	H5A—C5—H5B	109.5
C2—O1—H1	101.1	C4—C5—H5C	109.5
C8—N1—C9	115.8 (2)	H5A—C5—H5C	109.5
C2—C1—H1A	109.5	H5B—C5—H5C	109.5
C2—C1—H1B	109.5	C10—C6—C7	117.9 (2)
H1A—C1—H1B	109.5	C10—C6—S1	124.71 (17)
C2—C1—H1C	109.5	C7—C6—S1	117.38 (17)
H1A—C1—H1C	109.5	C8—C7—C6	118.8 (2)
H1B—C1—H1C	109.5	C8—C7—H7	120.6
O1—C2—C3	120.9 (2)	C6—C7—H7	120.6
O1—C2—C1	115.7 (2)	N1—C8—C7	124.6 (2)
C3—C2—C1	123.4 (2)	N1—C8—H8	117.7
C2—C3—C4	119.1 (2)	C7—C8—H8	117.7
C2—C3—S1	120.14 (18)	N1—C9—C10	124.5 (2)
C4—C3—S1	120.67 (18)	N1—C9—H9	117.7
O2—C4—C3	120.0 (2)	C10—C9—H9	117.7
O2—C4—C5	116.8 (2)	C6—C10—C9	118.4 (2)
C3—C4—C5	123.1 (3)	C6—C10—H10	120.8
C4—C5—H5A	109.5	C9—C10—H10	120.8
C4—C5—H5B	109.5		

### Hydrogen-bond geometry (Å, °)

**Table:**

*D*—H···*A*	*D*—H	H···*A*	*D*···*A*	*D*—H···*A*
O1—H1···O2	0.88	1.58	2.427 (3)	161
